# Assisted Knowledge Discovery for the Maintenance of Clinical Guidelines

**DOI:** 10.1371/journal.pone.0062874

**Published:** 2013-04-30

**Authors:** Emilie Pasche, Patrick Ruch, Douglas Teodoro, Angela Huttner, Stephan Harbarth, Julien Gobeill, Rolf Wipfli, Christian Lovis

**Affiliations:** 1 Division of Medical Information Sciences, University Hospitals of Geneva and University of Geneva, Geneva, Switzerland; 2 Bibliomics and Text-Mining Group, University of Applied Sciences Western Switzerland, Geneva, Switzerland; 3 Infection Control Program and Division of Infectious Diseases, University of Geneva Hospitals and Medical School, Geneva, Switzerland; Royal College of Surgeons, Ireland

## Abstract

**Background:**

Improving antibiotic prescribing practices is an important public-health priority given the widespread antimicrobial resistance. Establishing clinical practice guidelines is crucial to this effort, but their development is a complex task and their quality is directly related to the methodology and source of knowledge used.

**Objective:**

We present the design and the evaluation of a tool (KART) that aims to facilitate the creation and maintenance of clinical practice guidelines based on information retrieval techniques.

**Methods:**

KART consists of three main modules 1) a literature-based medical knowledge extraction module, which is built upon a specialized question-answering engine; 2) a module to normalize clinical recommendations based on automatic text categorizers; and 3) a module to manage clinical knowledge, which formalizes and stores clinical recommendations for further use. The evaluation of the usability and utility of KART followed the methodology of the cognitive walkthrough.

**Results:**

KART was designed and implemented as a standalone web application. The quantitative evaluation of the medical knowledge extraction module showed that 53% of the clinical recommendations generated by KART are consistent with existing clinical guidelines. The user-based evaluation confirmed this result by showing that KART was able to find a relevant antibiotic for half of the clinical scenarios tested. The automatic normalization of the recommendation produced mixed results among end-users.

**Conclusions:**

We have developed an innovative approach for the process of clinical guidelines development and maintenance in a context where available knowledge is increasing at a rate that cannot be sustained by humans. In contrast to existing knowledge authoring tools, KART not only provides assistance to normalize, formalize and store clinical recommendations, but also aims to facilitate knowledge building.

## Introduction

Antibiotics have been deployed massively over the last 70 years. Bugnon *et al.* [1] reported that up to 25% of patients admitted to the internal medicine and surgery wards in eight Swiss hospitals were treated with antibiotics. Several other studies [2]–[5] also concluded that more than a third of antibiotic prescriptions were unnecessary. Moreover, when an antibiotic was indicated, the specific treatment was considered incorrect in up to 65% [3] of the cases. Improving antibiotic usage has thus become a clear public-health priority. It is assumed that reducing the frequency and intensity of antibiotic use will result in waning antimicrobial resistance through a decrease in applied selection pressure.

The biomedical literature is the main source of knowledge for evidence-based practice. For antibiotic prescribing, different sources of biomedical literature are available: from primary sources (e.g. original studies) to secondary sources (e.g. summaries, syntheses) [6]. While original studies provide access to the most updated information, the reading and interpretation is time-consuming and requires highly-specialized expertise. Secondary sources of information are therefore interesting alternatives since they provide combined and processed information. However, they usually have a more limited coverage and are subject to bias, as well as delay in their generation.

A study conducted by Westbrook *et al.* [7] showed that using information retrieval systems to retrieve information from biomedical collections do help to improve medical prescriptions. Indeed, it is reported that clinical decisions were improved by 21% when clinicians spent up to six minutes interacting with information retrieval engines. The past years have seen the development of a wealth of search engines and text mining instruments to navigate the biomedical literature (e.g. GoPubMed, EBIMed, etc.). However, these information retrieval systems often generate high volumes of information at the expense of specificity and sensitivity to the query at hand: they provide the users with a set of relevant documents that need then to be manually processed. The success of such search engines to answer clinical questions is therefore limited. Indeed, Ely *et al.* [8] concluded that clinicians usually do not spend more than two minutes to search for an answer to a clinical question before giving up.

In this context, question-answering systems are an interesting alternative that directly extracts the information of interest out of these documents, thus facilitating the processing of such volumes of information. Competitions conducted by various organizations such as the Text Retrieval Conferences (TREC) [9]–[10] have greatly accelerated the development of open-domain question-answering systems, although domain-specific question-answering, such as medical question-answering, still lag behind [11]. Ben Abacha [12] described an approach based on the creation of semantic graphs. Documents and queries are first transformed into semantic graphs. Answers are obtained by matching between the graph of the query and the graphs of documents. Terol [13] described a question-answering system relying on the application of complex natural language processing techniques to infer the logic forms. This system focuses on ten types of medical questions. More general question-answering systems [14], such as AskHERMES [15]–[16] or HONqa [17], are based on a robust semantic analysis to answer complex clinical questions, using various resources such as MEDLINE, eMedicine and Wikipedia.

Biomedical literature is only one of the numerous sources of information that need to be taken into account to prescribe relevant antibiotics. Handling all the sources of information (e.g. microbiological data, health costs) at the point-of-care is extremely tedious and does not always lead to the most optimal choice. Clinical practice guidelines (CPGs) enable to synthesize the voluminous amount of available information, thus allowing physicians to easily and rapidly access to high quality recommendations relying on the principle of evidence-based medicine. However, the development of evidence-based CPGs is a complex process.

A variety of guidelines support tools has been designed for assisting the development and management of CPGs. The most simplistic approaches are directed toward formalization of already existing guidelines, such as G-DEE [18], a document-centric approach to facilitate the structuring of free-text guidelines using a set of XML mark-ups. The Guideline Markup Tool [19], developed within the Asgaard project, aims to facilitate the translation from unstructured HTML guidelines into the formal representation Asbru. Other approaches go a step further and provide an editor to directly create the recommendations. The HELEN-Project [20] is a modular framework for representing and implementing CPGs. A guideline editor, based on Protégé-2000, allows creating and editing guidelines according to a specific ontology (“HELEN Ontology”) and stores the guidelines in Protégé-2000 format or XML. Dunsmuir *et al.* [21] designed an authoring tool to help anaesthesiologists to easily encode their knowledge that will then be used in a rule-based decision support system. The user interface allows the clinicians to easily fill each part of the rule. Finally, the Guide Project [22] not only provides an editor to create recommendations, but also aims to improve guideline sharing among health care organizations by providing a common repository that could be adapted and revised at local levels across organizations. However, none of these support tools take into consideration the acquisition of clinical evidences. The systematic reviews of the literature that are required to extract evidences are a particularly labor-intensive and time-consuming step [23].

The Detecting and Eliminating Bacteria UsinG Information Technology (DebugIT) project [24] is a FP7 European initiative aiming to improve antibiotic usage through information technology. To achieve DebugIT’s objectives, clinical data are first collected from seven European clinical centers and stored in a distributed Clinical Data Repository (CDR) [25], [26]. Text-mining and data-mining methods are then applied to acquire new knowledge that is stored in a Medical Knowledge Repository (MKR). Finally, this knowledge is used to provide better-quality health care through different clinical applications, such as decision support and trend monitoring [27]. The MKR is deployed in each participating clinical center and can be shared within the DebugIT Linked Data infrastructure.

In the context of the DebugIT project, we propose a computer-assisted approach to improve the management of CPGs. We consider the development of a Knowledge Authoring and Refinement Tool (KART), with the aim to facilitate the authoring and maintenance of clinical guidelines knowledge on three levels: building, implementation and dissemination. In this paper, we consider a clinical recommendation as a simple statement complying with the following logical pattern: “an antibiotic A is used to treat a disease D caused by a pathogen P under clinical conditions C”. First, we investigate the development of a highly specialized question-answering engine to accelerate the search for clinical knowledge from various scientific libraries, such as MEDLINE. Therefore, we aim to facilitate the building of CPGs. Second, we explore the simplification of the implementation of CPGs by using automatic text categorizers able to recognize domain-specific entities, such as drugs. Third, we examine the question of the dissemination and sharing of CPGs, by the use of automatic formalization procedures to store recommendations in an online repository. This paper focuses on presenting the design and evaluation of KART.

## Methods

In this section, we describe the design of the three main modules of KART and report on the methods employed to assess the usability and utility of KART.

### Module 1: Medical Knowledge Extraction

In this module, we design a specialized search engine dedicated to ease the acquisition of antibiotherapy data for the creation of evidence-based CPGs, in order to improve antibiotic stewardship. We aim to automatically extract antibiotic treatments from online scientific libraries. We functionally describe this task as a question-answering problem, corresponding to the following pattern: “What antibiotic A treats a disease D caused by a pathogen P?”. Answers are retrieved by a specialization of the EAGLi question-answering engine [28]–[30]. Strategies to specialize the EAGLi’s Application Programming Interface to obtain more optimal answers have been described elsewhere [31]–[33]. We report here the final customization of the system.

Tuning and evaluation of this specialization is based on a benchmark of 72 recommendations manually extracted and translated from the therapeutic guide of major infections in elderly patients edited and provided by the Antibiotic Stewardship Program of the University Hospitals of Geneva (HUG), a 2000-bed consortium of 8 public and teaching hospitals in the canton of Geneva, Switzerland. This guide is delivered as a 36-page MS-Word document written in French. For each recommendation, up to three antibiotics are proposed. The objective is to retrieve the recommended antibiotics given the parameters of the recommendation (i.e. disease+pathogen+clinical conditions). Twenty-three recommendations are used for tuning the system, while the 49 remaining recommendations are used to evaluate the final system settings. Our experiment is considered as an information retrieval task, or more precisely a factoid QA task. Thus, we focus on precision-oriented metrics. In particular, the precision of the top-returned answer (so-called P0 or mean reciprocal rank [34]) is used to evaluate the effectiveness of our approach. This metric reflects the ability of a system to find the relevant answers on the top of the ranked list of answers. We tune the system to maximize P0. To complement this metric, which provides the precision of a system, where the user would ignore answers provided in position 2 or lower ranks (i.e. a fully automatic system with no user interaction), we also measure the recall of the system achieved by the top five answers (i.e. R5). This metric reflects the ability of a system to find a maximum of relevant answers in the top five answers. Thus, we try to estimate how useful such a system would be when used by an expert able to validate the ranked output of the guideline generator. The metrics are obtained using TrecEval, a program developed to evaluate TREC results using US National Institute of Standards and Technologies (NIST) evaluation procedures. Statistical differences among runs are assessed using a two-tailed randomization test with a 1% confidence level.

The first step of the question-answering process is an information retrieval task, in which a set of relevant documents is retrieved from a broader collection. Tuning of this step can be done at three levels: the collection of documents, the retrieval engine and the number *k* of retrieved documents. Online scientific libraries are an important source of knowledge to assist physicians in their daily practice. For instance, it has been shown that MEDLINE contained relevant information for answering more than half of the clinical questions posed by primary care physicians [35]. In the KART framework, three collections are tested: MEDLINE, which contains about 19 millions citations of biomedical journals; PubMed Central, an online database of more than 2.4 millions full-text scientific articles in health and life sciences; and the Cochrane Library, a database of systematic reviews and meta-analyses in medicine. Two search engines are tested: PubMed, the National Library of Medicine’s Boolean search engine developed by the NCBI, which ranks documents chronologically; and EAGLi’s search model, which uses a vector-space retrieval engine; see Singhal [36] for an introduction to information retrieval. Ultimately we also evaluate the effectiveness of a meta-search engine, which linearly combines the results provided by each search engine [37]. The optimal number of documents to be retrieved to compute the answers is also tuned.

The second step is an information extraction task, where candidate answers are extracted from the returned set of documents. Tuning of this step addresses two levels: the target terminology and the number *n* of retrieved answers per query. Answer extraction relies on a target terminology, which lists all possible answers for a given semantic type (e.g. antibiotics). Terms from this list are mapped to the retrieved documents through pattern-matching strategies allowing minor morphological variations such as plural forms. Target terminologies are usually constructed using existing controlled vocabularies, such as the Medical Subject Headings (MeSH). In our case, a subset of the Anatomical Therapeutic Chemical Classification provided by the World Health Organization (WHO-ATC), corresponding to the branch *J01*, is used as the basis of our target terminology. This target terminology (T1) consists of 266 antibiotics, with a preferred term and an identifier for each antibiotic. Two variants are created; the first one (T2) enriched with synonyms provided by the MeSH and the second one (T3) with the use of more common terms, manually defined, for the combined antibiotics (e.g. *amoxicillin and enzyme inhibitor* is replaced with *amoxicillin clavulanate*). Finally, the optimal maximum number of answers to be retrieved for each query is also determined.

The third step aims to improve the retrieval effectiveness of the search engine by filtering out irrelevant documents. Despite PubMed proposes a set of methodological search filters, we did not use it since only the PubMed search engine would be able to use it, while alternative search engines, in particular the EAGLi search engine, do not assume such a user-specific interaction. Instead, we rely on various metadata attached to publications. This strategy is performed only for the MEDLINE and PubMed Central collections, since these metadata are not available for the Cochrane Library. We perform various tuning based on the combination of four parameters in order to exclude documents deemed irrelevant to our task. First, we focus on the publication date of the documents. It is indeed a well-known fact that CPGs must evolve with time due to the apparition of bacterial resistances. Thus, old publications are of little relevance for our task since they might recommend antibiotics that should not be used anymore. We therefore assume that excluding old publications should improve the effectiveness of our system. Different time frames are tested (e.g. past five years). Second, we focus on the language of the publications. It was reported by infectious disease specialists that the publications in “exotic” language were of little use as they were unlikely to be understood by the average user in Western Europe or Northern America. We thus perform an experiment where we exclude non-English publications from our set of relevant documents. Third, we explore the impact of the publication type. Obviously, some publication types have a higher interest than others for CPGs. From prior discussions we had with local experts, it appeared that reviews and clinical treatment guidelines are considered of major importance. In contrast, case reports are regarded as of little use for clinicians seeking guidance with the most evidence-based approach to a common infection or clinical scenario since they often report on rare infections or rare presentations. Two strategies are tested: to exclude a publication type (i.e. case reports) or to limit to some publication types (i.e. reviews or practice guidelines). Fourth, we wonder whether the use of MeSH terms attributed to publications may be used to filter out non-relevant publications. For this setting, we test the presence of different MeSH terms, such as *Humans*, *Anti-bacterial agents*, *Therapeutic use* or *Drug Therapy* in the MEDLINE notice.

The final step ranks the candidate answers by relevance to the query at hand. Our strategy is based on the redundancy principle [28]. Indeed, we assume that, given the large amount of data contained in scientific libraries like MEDLINE, a correct answer should be found in several documents and usually several times in these documents. Ranking is based on scores attributed to each concept from the target terminology, calculated based on both the frequency of this concept in each document and the frequency of documents containing this concept. For the vector-space search, the relevance score assigned to each document is also used.

Assuming that the selection of an antibiotic by a physician depends on several dimensions not yet captured by our relevance-driven model, we then performed three additional experiments to improve ranking of answers by directly injecting statistical information derived from the HUG Computerized Physician Order Entry (CPOE). Here, we focus on the following three types of information: antibiotic cost, resistance profiles and adverse drug reactions.

If two treatments lead to the same outcome and have similar benefits and harms, the less expensive compound should be preferred. Thus, we re-rank the list of antibiotics obtained previously in such a way that more expensive compounds are ranked lower, while less expensive antibiotics are ranked higher. This experience is based on two different lists of antibiotic costs: the costs of 129 products in 2009 provided by the pharmacy of the HUG and the costs mentioned in the Swiss Compendium of Drugs. We first calculate the cost of one day of treatment, using respectively prescription data of the HUG to obtain the number of daily doses usually prescribed for each product and dosage information mentioned in the Swiss Compendium of Drugs. We then merge all products corresponding to the same pharmaceutical substance. Finally, an arbitrary cost is defined for antibiotics absent from our lists. This cost is fixed during the tuning phase by determining the less penalizing arbitrary value.

Performing a microbiological analysis before initiating antibiotic therapy is the optimal way to prescribe an antibiotic to which the pathogen is sensitive. Thus, we use resistance profiles to promote antibiotics with low resistance levels and thus relegate antibiotics to which the specific pathogen has shown high resistance. This experience uses current data from antibiograms stored in the HUG’s CDR. Assuming that guidelines to treat bacterial infections are normally not time-specific, i.e. the recommendation for a prescription of an antibiotic is the same during all the year, we decided to work on a (modulo) 12-month frame to neutralize seasonal biases. Three time frames are tested: resistance profiles in 2006, in 2007 and finally in both 2006 and 2007. Antibiograms are extracted from the CDR using Simple Protocol and RDF Query Language (SPARQL) queries for each pair of pathogen-antibiotic. Results are then parsed to compute a susceptibility score, corresponding to the percentage of antibiograms where a susceptibility outcome was observed out of the whole antibiograms performed for the given pair. Finally, an arbitrary resistance value is defined for pathogen-antibiotic pairs absent from the CDR. This value is set up during the tuning phase by determining the less penalizing arbitrary value.

Similarly, we hypothesize that adverse drug reactions should opportunely be taken into account by our system. Indeed, a drug causing serious adverse effects should be avoided if an alternative treatment causing less harm exists. Our strategy to extract adverse drug reactions relies on the data provided by DrugBank. Each antibiotic is classified in one of the following classes: 1) no adverse drug reaction reported; 2) moderated adverse drug reactions reported; and 3) severe adverse drug reactions reported. The distinction between moderate and severe adverse drug reactions is based on a set of regular expressions. We defined a list of terms considered as severe (e.g. death, coma, dangerous). The presence of one of this term in the *toxicity* field implies the classification of the drug in the third category. Finally, a default value is assigned to antibiotics absent from DrugBank. To set up the optimal default value, we perform several runs, each with a different default value, and select the value resulting in the highest top-precision.

### Module 2: Normalization of Clinical Recommendations

Normalization of clinical recommendations attempts to attribute unambiguous descriptors to the different parameters of the recommendations. For KART, the following terminologies have been chosen: the Tenth Revision of the International Classification of Diseases (ICD-10) for diseases, the New Taxonomy database (NEWT) for pathogens, the WHO-ATC terminology for antibiotics, and the Systematized Nomenclature of Medicine - Clinical Terms (SNOMED-CT) for any additional clinical conditions (e.g. pregnancy, age-related groups, etc.).

Our approach consists of a semi-automatic normalization. We use online automatic text categorizers, such as SNOCat [38] for the SNOMED-CT terminology, which are hybrid systems based on both regular expressions and vector-space methods to associate concepts to an input text. Given a term or an expression, the categorizer proposes a list of relevance-ranked concepts. The user must then select the concept judged as the most relevant to represent the entity of interest. For the NEWT taxonomy, we rely on a dictionary-based strategy combined with simple rules. When the user tries to normalize a species not available in NEWT, our approach will suggest the class to which this species belongs.

### Module 3: Formalization and Storage of Clinical Recommendations

Most CPGs are published in free text (HTML, PDF, etc.), which is a major problem when we aim at implementing those guidelines in the clinical decision support system (CDSS) of an Electronic Health Record (EHR). Formalization of recommendations is thus a crucial step to allow automatic machine-interpretation of the recommendations [19]. There are numerous available formalisms, such as Asbru [39] or Guideline Interchange Format (GLIF) [40]–[42]. We use Notation-3, which is a non-XML serialization of Resource Description Framework (RDF). Thus, this formalism can translate any representation of the semantic web. This choice was guided by the industrial options done within the DebugIT project, under the coordination of Agfa Healthcare. A Java web service automatically performs the conversion in the MKR’s SPARQL endpoint where the recommendation is stored. Previous versions of the recommendations are also archived via the creation of RDF documents. Each document contains the status of the recommendation (e.g. modified, obsolete).

### User Assessment

The clinical validation of the KART system was conducted at HUG. The evaluation was based on two dimensions: the utility and the usability of the system. The practical usefulness of the system is ensured only if the system is utile and usable. Utility informs about the usefulness for the user of the functionalities provided by the system. Usability refers to how easy these functionalities can be employed. We choose to evaluate KART using the method of the cognitive walkthrough [43]–[45]. This method consists of an exploration of the system by evaluators who follow a sequence of actions defined during the preparatory phase. The evaluators report on the problems encountered. The cognitive walkthrough is typically performed by domain or usability experts who were not involved in the development of the system. Software engineers are usually not the end-users and therefore cannot evaluate whether or not the content provided by KART is relevant for the given work context. In addition, it is difficult for them to predict whether the functionalities are easy to use and consequently whether the system will be accepted. For instance, the automatic extraction of antibiotic treatments from the literature may be a valuable functionality since it facilitates the access to literature and decreases the process time required. However, if the quality of the content returned by this functionality is poor, the automatic extraction becomes a worthless functionality and will be rejected. In order to test the utility and usability, an independent team of clinical infectious disease specialists reviewed the KART system by the means of ten clinical scenarios (e.g. *community-acquired pneumonia*; *cystitis in a pregnant woman*). For each of these scenarios, the evaluators walked through the entire process of creating a recommendation using KART. The resulting data are a set of findings and propositions for further improvement of the utility and usability from the standpoint of clinical experts.

The main functionality – the medical knowledge extraction – of KART was evaluated at three levels. First, for each of the clinical scenarios, the top five antibiotics returned by KART were compared to the top five recommended antibiotics. Recommended antibiotics were based on the local CPGs of the HUG as well as those provided by both Uptodate, Inc., and the Infectious Disease Society of America, two organizations whose recommendations are followed worldwide in clinical infectious diseases. Second, the relevance regarding the clinical case and the suggested antibiotic of the top five publications proposed by KART for the most-cited antibiotic was analyzed. This analysis is based on the subjective opinion of the evaluators, who are considered in this study as “gold standards”. Finally, an overall score was assigned to evaluate both the recommended antibiotics and the literature provided with the following scale: (1) potentially harmful (the antibiotic recommended would not only be inappropriate for the clinical query at hand, but may be harmful to the patient), (2) of dubious relevance to the clinical query at hand, (3) acceptable and (4) excellent.

## Results

In this section, we first present the implementation of KART. Second, we describe the quantitative results obtained by the medical knowledge extraction module to automatically extract antibiotic treatments from literature. Third, we report on the results of the user-based evaluation of KART.

### Implementation

KART is implemented and deployed as a standalone web application. The web application is developed using Adobe Flash Builder 4, a software development kit for the development and deployment of Rich Internet Applications based on the Adobe Flash platform. The web-services are developed using the Java platform Enterprise Edition version 5, and are deployed in Apache Tomcat version 5.5. The Jena open source Semantic Web Framework is used for querying the MKR, a generic semantic repository created by Agfa Healthcare within the DebugIT project.

**Figure 1:**
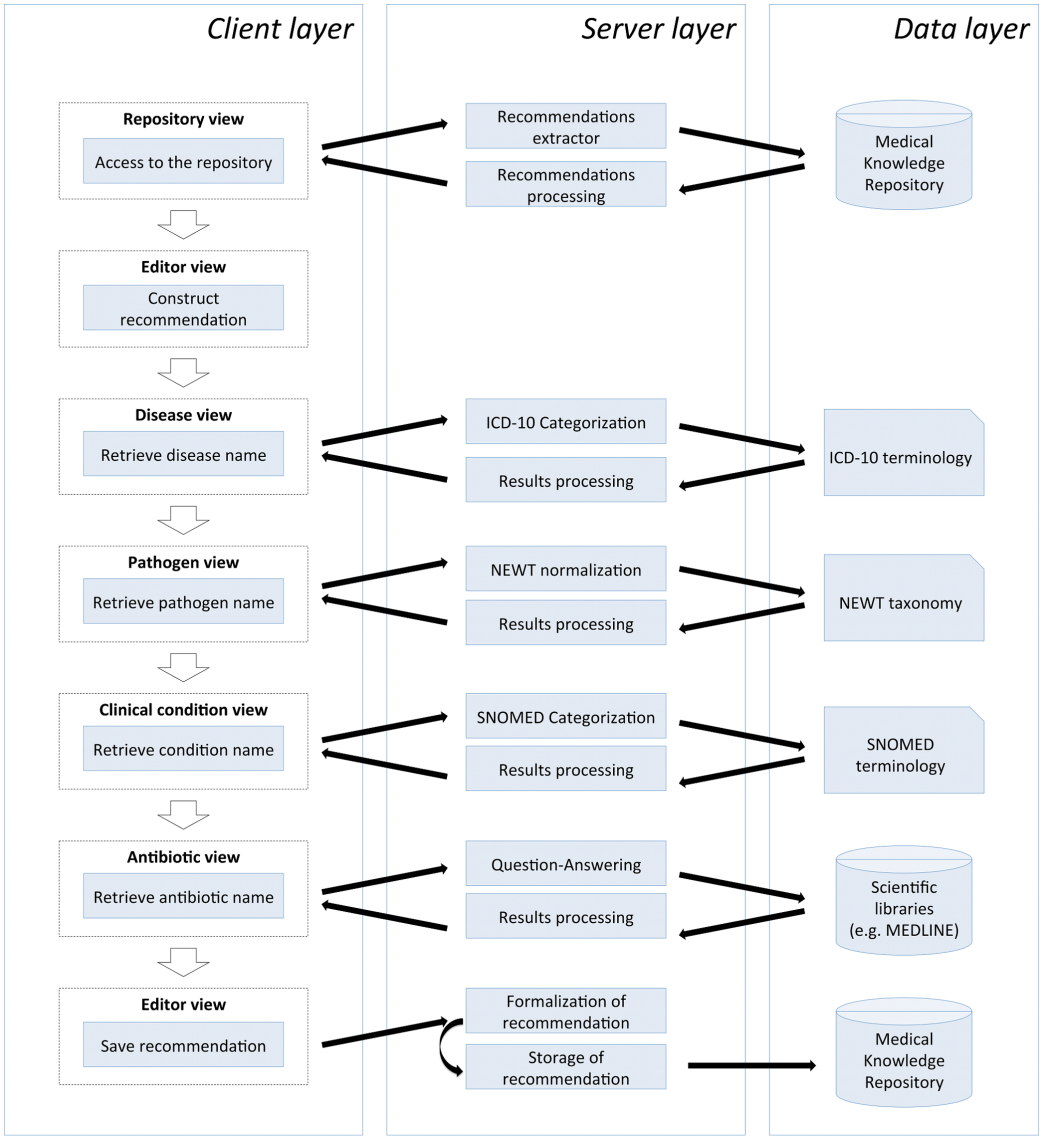
System architecture of KART. On the client side, a repository containing all existing recommendations is first presented. Then, a module to edit, formalize and store the recommendation is presented. Finally, KART offers modules for the acquisition of normalized data for the parameters of the recommendation. On the server side, Java web services communicate with the MKR through SPARQL queries to extract and store recommendations. They are able to transform recommendations from Notation-3 to human-readable format and vice versa. We also propose Java web services to acquire normalized data by querying existing categorizers and a question-answering engine. On the data side, the generic semantic repository MKR is accessed. Several terminologies are necessary for categorization and normalization, and scientific libraries are used by the question-answering engine.

**Figure 2:**
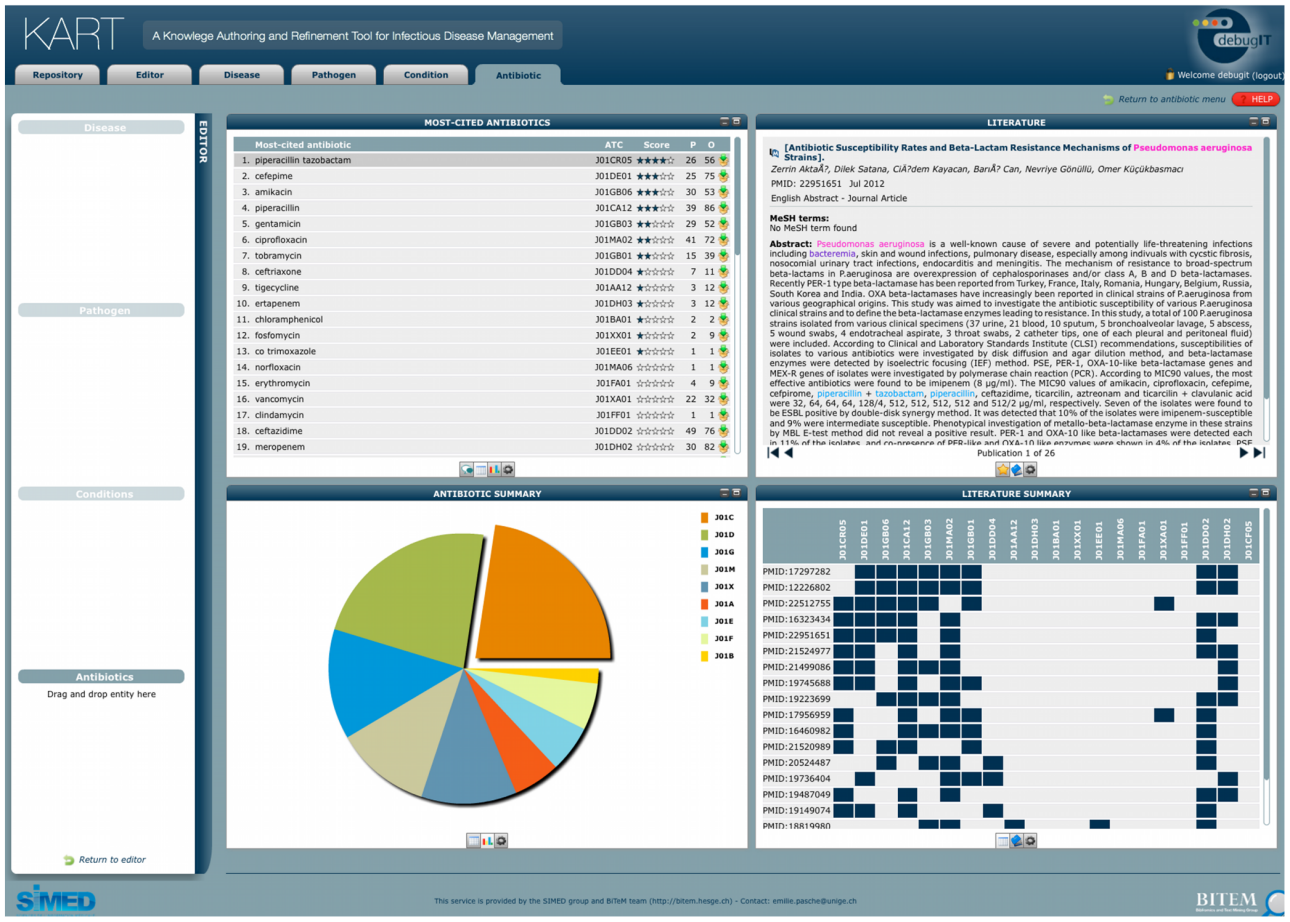
Example of the design of KART for the automatic generation of antibiotic treatment. A literature search has been performed to find treatments to treat *bacteremia* caused by *Pseudomonas aeruginosa*. The output is divided into four panels: 1) on the top-left panel, a ranked list of the most-cited antibiotics in literature is proposed; 2) on the bottom-left panel, an aggregation of these antibiotics is displayed to determine the main classes of antibiotics involved; 3) on the top-right panel, the publications supporting each antibiotic are displayed; and 4) on the bottom-right panel, an alternative way to process the literature is provided: a list of the twenty most frequent publications found by the question-answering module is displayed and shows which of the twenty first-ranked antibiotics are present in each of these publications.

KART is accessible at http://eagl.unige.ch/KART2 and is designed according to the architecture presented in Figure 1. This architecture follows the process of constructing a clinical recommendation. Once logged in, users first access the repository of existing recommendations. Three actions are proposed: browsing/filtering the repository, editing recommendations and creating new recommendations. The edition/creation of recommendations takes place in the KART editor, where three actions are available: managing parameters of a recommendation (i.e. disease, pathogen, antibiotic and clinical condition), formalizing the recommendation in a Linked Data format and saving the recommendation in the MKR. The management of the parameters is supported by modules to facilitate the acquisition of normalized data using standard terminologies: 1) the user enters any free text and retrieves a list of relevant concepts corresponding to the input; not available for antibiotics; 2) the user enters the correct concept and its terminological identifier that are verified by the system; 3) the user formulates a question to the system and obtains a list of candidate answers (Figure 2); only available for antibiotics.

### Module 1: Medical Knowledge Extraction

Tuning of the information retrieval parameters is presented in Table 1. The MEDLINE collection obtains the best results, followed closely by the PubMed Central collection. The Cochrane Library presents the less effective results, with the lowest top-precision and recall at 5. Only the vector-space model is available to search the content of the Cochrane Library. We observe that the two search engines behave differently. The PubMed search engine improves the results compared to the vector-space search engine of, respectively, +59.4% (p<0.01) for the MEDLINE collection and +8.1% (p<0.01) for the PubMed Central collection. However, we also observe that the PubMed search engine is able, in both cases, to answer fewer queries compared to the vector-space search engine. As expected, the combination of both search engines achieves better effectiveness, providing answers to all queries with an overall improvement of the top-precision compared to the vector-space engine for the MEDLINE collection (+17.8%, p<0.01) and the PubMed Central collection (+9.4%, p<0.01). Similarly, increasing the size of the set of documents retrieved for the MEDLINE collection results in a higher top-precision (optimal *k* = 300). The optimal *k* values for PubMed Central (*k* = 40) and the Cochrane Library (*k* = 4) are much lower, which is consistent with the size of the respective collections.

**Table 1:** Tuning of the information retrieval parameters for the Question-Answering task.

Collection	Search engine	Number of documents	Queries answered	P0	R5
MEDLINE	Vector-space	50	22/23	0.36	0.23
		300	23/23	0.47	0.29
	PubMed	50	13/23	0.57	0.44
		300	13/23	0.61	0.44
	Combination	50	23/23	0.42	0.28
		300	23/23	0.47	0.30
PubMed Central	Vector-space	50	23/23	0.35	0.20
		40	23/23	0.37	0.20
	PubMed	50	13/23	0.38	0.26
		40	14/23	0.38	0.33
	Combination	50	23/23	0.38	0.22
		40	23/23	0.40	0.22
Cochrane Library	Vector-space	50	22/23	0.20	0.15
		4	22/23	0.30	0.20

The fine-tuning of the information extraction parameters for MEDLINE is presented in Table 2. Increasing the number of answers to be extracted from the documents has no impact on the results. The choice of the target terminology has a higher impact on the system’s precision. We thus observe that the use of synonyms results in a significant decrease (–18%, p<0.01) of the top-precision. However, the use of more common terms to describe combined antibiotics shows a modest improvement of the top-precision (+1.6%, p<0.01). Similar results have been observed for the other collections.

**Table 2:** Tuning of the information extraction parameters for the Question-Answering task.

Collection	Target terminology	Number of answers	Queries answered	P0	R5
MEDLINE	T1	20	23/23	0.47	0.30
	T2	20	23/23	0.39	0.33
	T3	20	23/23	0.48	0.27
	T3	100	23/23	0.48	0.27

The tuning of the filters based on the metadata is shown in Table 3. Regarding the publication date, the best results are obtained when the limit is set up to the past 15 years for the MEDLINE collection (+4.2%, p<0.01) and to the past 5 years for the PubMed Central collection (+1.0%, p<0.01), which contains relatively more recent articles. Filtering documents not published in English has not improved our results. We even observe a strong decrease of the top-precision for the MEDLINE collection (–20%, p<0.01). We therefore assume that these abstracts contain relevant information that may be successfully interpreted by our QA engine, even though the non-English publications are regarded as useless for experts of infectious diseases, who are mainly interested in full-text contents. No change is observed for the PubMed Central collection, which is not surprising since PubMed Central contains only English publications. On the publication type level, the best results are obtained when case reports are filtered out. It results in a moderate improvement of the top-precision for MEDLINE (+1.8%, p<0.01) and for PubMed Central (+6.3%, p<0.01). The use of the MeSH terms assigned to publications allows improving results for the MEDLINE collection only, suggesting that looking for MeSH descriptors in full-text is much less effective than MeSH assigned by librarians of the National Library of Medicine. A significant improvement of the top-precision (+1.9%, p<0.01) is obtained only when documents containing the MeSH term *humans* are selected, thus excluding veterinary studies, where antibiotherapy complies with different guidelines. Finally, the combination of all best tuned parameters mentioned above results in a significant improvement of the top-precision for both the MEDLINE collection (+11%, p<0.01) and the PubMed Central collection (+8.1%. p<0.01). MEDLINE is filtered to retrieve only documents published after 1997 that are not case reports and that mention the descriptor *Humans* in the MeSH terms section. The PubMed Central collection is filtered to retrieve only documents published after 2007 that are not case reports.

**Table 3:** Tuning of the publications filters for the Question-Answering task.

Collection	Filters	Queries answered	P0	R5
MEDLINE	No filter	23/23	0.48	0.27
	Date filter: limit to the last 15 years	23/23	0.50	0.34
	Language filter: limit to English publications	23/23	0.38	0.29
	Publication type filter: exclude case reports	23/23	0.49	0.33
	MeSH term filter: limit to publications containing *humans*	23/23	0.49	0.28
	Combination of filters	23/23	0.53	0.34
PubMed Central	No filter	23/23	0.41	0.22
	Date filter: limit to the last 5 years	23/23	0.41	0.20
	Language filter: limit to English publications	23/23	0.41	0.20
	Publication type filter: exclude case reports	23/23	0.43	0.22
	Combination of filters	23/23	0.44	0.20

Tuning of the re-ranking experiments is presented in Table 4. Cost inclusion shows quite promising results for all collections with a respective improvement of the top-precision of +17.0% (p<0.01) for MEDLINE, of +9.2% (p<0.01) for the PubMed Central collection and of +51.0% (p<0.01) for the Cochrane Library. Resistance inclusion produces contrasting results. The 2006 time frame performs the best with all collections. MEDLINE and the Cochrane Library positively benefit from the resistance inclusion with an improvement of, respectively, +4.6% (p<0.01) and +10.4% (p<0.01). In contrast, PubMed Central undergoes a slight degradation of top-precision of –0.4% (p<0.01). Similarly, the inclusion of adverse drug reaction produces contrasting results. No improvement (–5.9%, p<0.01) of the top-precision was observed when the re-ranking of the results was performed for the MEDLINE collection. A slight improvement was observed for both the systems based on the PubMed Central collection (+4.6%, p<0.01) and the Cochrane Library (+2.5%, p<0.01). Finally, when including several modalities, we observe that despite a significant improvement of the top-precision for both MEDLINE (+12.1%, p<0.01) and the Cochrane Library (+17.0%, p<0.01), the re-ranking based on the single antibiotic costs information is performing better. In opposite, the use of the PubMed Central collection results in a positive impact (+14.5%, p<0.01) when the re-ranking is based on both drug costs and adverse drug reactions.

**Table 4:** Tuning of the re-ranking experiments for the Question-Answering task.

Collection	Re-ranking	Queries answered	P0	R5
MEDLINE	Baseline	23/23	0.53	0.34
	Cost (based on Swiss Compendium)	23/23	0.62	0.38
	Resistance (based on data from 2006)	23/23	0.56	0.37
	Adverse Drug Reaction	23/23	0.50	0.32
	Combination	23/23	0.60	0.33
PubMed Central	Baseline	23/23	0.44	0.20
	Cost (based on HUG cost list)	23/23	0.48	0.34
	Resistance (based on data from 2006)	23/23	0.44	0.32
	Adverse Drug Reaction	23/23	0.46	0.25
	Combination	23/23	0.51	0.27
Cochrane Library	Baseline	22/23	0.30	0.20
	Cost (based on HUG cost list)	22/23	0.46	0.30
	Resistance (based on data from 2006)	22/23	0.33	0.30
	Adverse Drug Reaction	22/23	0.31	0.19
	Combination	22/23	0.43	0.22

The final tuning applied on the evaluation benchmark is presented in Table 5. The best results are obtained with MEDLINE, which is able to answer correctly to more than half (53%) of the queries. For comparison, the same set of queries obtained a top-precision of only 20.4% when the initial non-specialized question-answering engine was used on the MEDLINE collection, thus showing a significant improvement of +155% of the specialized version. The Cochrane Library obtains a top-precision of 47%, but is limited by the ability to answer to only 41 questions out of the 49. The PubMed Central collection obtains less effective results, with a top-precision of 31% and the ability to answer to 48 questions out of 49.

**Table 5:** Final results of the medical knowledge extraction module.

Collection	Re-ranking	Queries answered	P0	R5
MEDLINE	Baseline	49/49	0.53	0.29
PubMed Central	Baseline	48/49	0.31	0.20
Cochrane Library	Baseline	41/49	0.47	0.30

### User Assessment

The user-based evaluation of the medical knowledge extraction module showed that for five clinical scenarios, at least one of the top five recommended antibiotics were found in the top five ranked antibiotics proposed by KART, which is consistent with our quantitative evaluation. Concerning the literature provided by KART to support suggestions, only three publications out of the 45 examined were reported as being of high interest for clinicians, 10 were of moderate interest, while the rest was of little or no interest. Main reasons for the absence of interest included the fact that publications were not in English – many English abstracts in MEDLINE are not provided with a full-text article in English – or were outdated – the EAGLi search engine ranks publications by relevance to the query and does not take into account the publication date. As an example, the top returned publication supporting *gentamicin* in case of *streptococcal endocarditis* in *adult* was judged of little interest because it was a case report concerning a specific patient. In opposite, the third publication was judged of high interest because it was a prospective comparative study. It is to be noted that the search engine used at the time of the evaluation did not include the filtering of irrelevant publications described in the module 1. A manual analysis of the literature provided by PubMed with the same clinical scenarios showed that the literature provided by KART was generally more relevant. Finally, KART recommendations for six clinical scenarios received the acceptable tag, while two were deemed to be of doubtful relevance and two others were judged as potentially harmful.

The evaluation of the KART’s ability to normalize clinical recommendations showed that, despite it being indispensable for the creation of a machine-readable recommendation, this module had a rather mixed interest for end-users. The major problem highlighted was the difficulty in finding a relevant concept to represent the clinical term specified by the user. Indeed, it frequently happened that the proposed concepts were either too general or too specific (e.g. when searching for *brain abscess*, only general concepts such as *brain, unspecified* or overly specific concepts such as *amoebic abscess of the brain* were proposed). Therefore, the user often had to capture different descriptions before being able to select a satisfactory concept. The limited expression power of standard terminologies is a known problem when capturing controlled information in pre-defined medical templates, such as the EHR.

Unfortunately, the last component of the system (i.e. the clinical recommendation formalization and storage module) could be assessed neither quantitatively nor qualitatively as the CDSS able to operate with such formalisms has yet to be developed. Nevertheless, the evaluation of KART by clinical experts in infectious diseases has shown that displaying the formalized recommendation is probably not necessary and sometimes even confusing, since it produced codes with which clinicians are not familiar. It was thus recommended to hide formalization in the next release.

It is also important to mention that this evaluation was based on a small number of evaluators. For this sake, the remarks and opinions mentioned in this report represent solely the evaluators’ point of view and cannot be extrapolated to the community of infectious disease specialists.

## Discussion

In the context of this paper, we were interested in the process that leads to the development of CPGs. Such guidelines aim to facilitate access to high quality information at the point-of-care in order to reduce inappropriate prescriptions of antibiotics. While in principle we could have thought that the rate of inappropriate prescriptions would be drastically reduced – and thereby the selection pressure would be reduced – de facto, it appears that CPGs do not get the desired effects [46], [47]. Several studies have highlighted the limitations of these guidelines, restraining the application of the recommendations carried by the CPGs. These limitations affect each of the three main axes of the lifecycle of the CPGs management: the development (e.g. the large quantity of available data makes difficult the creation and updating of CPGs), the dissemination (e.g. many CPGs are developed at a local scale and are not openly shared among institutions) and the implementation (e.g. the lack of standards makes difficult the machine interpretation of the CPGs).

In this complex context, we have developed an innovative tool for creating, validating and maintaining clinical recommendations for antibiotic prescriptions. Thus, we have sought to improve the full lifecycle of the CPGs management with respect of each of the problems mentioned earlier. Although further improvements are needed to make KART fully usable for the targeted users (i.e. the infectious disease specialists who develop and maintain CPGs), the system contrasts with existing knowledge authoring tools, because it not only aids in the normalization, formalization and storage of clinical recommendations, but also facilitates knowledge acquisition by the institution. The database of recommendations created and validated by KART can then be imported to any electronic prescription system able to read Linked Data. At a final stage, the so-created CPGs can be used by CDSSs and help physicians to improve antibiotic prescribing practices.

One of the critical steps in the creation of these guidelines is the need to perform a systematic review of the literature, which is an extremely time-expensive and costly procedure, because of the necessity to collect all the evidences relevant to a given subject. Therefore, we have proposed an innovative method to acquire these evidences. Instead of the traditional search engine that returns a large number of documents that users have to browse one by one to find the information of interest, we have proposed an approach that directly extracts the information of interest out of these documents. This module, which is the main feature of KART, is a promising alternative to state-of-the-art information retrieval systems since it processes the literature and rapidly provides a ranked list of answers, thus facilitating the access to pertinent information on antimicrobial treatments. Beyond marketed initiatives [48], KART modestly provides an innovative way to browse the literature: publications are clustered by treatments and most-cited treatments are ranked higher, thus facilitating the systematic reviews of the literature. More than half of the clinical queries entered into KART received a correct answer in the top position, while the non-specialized question-answering system only allowed finding a correct answer in the top position for a fifth of the queries. Therefore, our different experiments of customization helped to greatly improve the performance (+155%). Although our performances remain modest, it must nevertheless be pointed out that it is unrealistic to hope to achieve an accuracy of 100%. Indeed, different users have different needs and expectations. It has been showed that for question-answering tasks, two assessors had an agreement of only 84%, regarding what they consider as a correct answer. This results drop down to 64% when three assessors are taken into account [9], [49]. In other words, if a given user considers that a system answers correctly to all of his questions, only 84% of the answers will be considered as satisfactory for another user.

It must be stressed that KART is not a decision-making tool. This search engine is dedicated to facilitate CPGs building by infectious disease specialists, and does not intend to be directly used by physicians at the point-of-care. Indeed, our experiments showed that “only” half of the treatments proposed by KART in the top position are compliant with clinical practice guidelines; others being second-line treatments or even sometimes erroneous treatments. Two main categories of queries were particularly problematic for our approach. In some infectious disease scenarios, antimicrobial therapy is in fact contraindicated. For instance, the *diarrhea* caused by *enterohaemorrhagic Escherichia coli* in *children* should not be treated with any antibiotic, since it can induce the *hemolytic-uremic syndrome*. Our tool is not able to cope with this nuance of clinical medicine. Indeed, KART’s statistical answering model relies significantly on the frequency of citations and is currently not able to interpret the occurrence of negations in the text. Thus, a large amount of literature retrieved by the search engine and recommending avoiding a given antibiotic will be misinterpreted. Another problem is the necessity in some cases to prescribe a combination of several antibiotics. While some combinations are defined in terminologies, such as *amoxicillin and piperacillin*, others are not. Since KART is only able to provide antibiotics that are in our target terminology, it is often unable to propose combined antibiotherapies. However, KART associates literature with each of its suggestions and the careful reading of these publications combined with clinical expertise should enable the validation or the exclusion of a suggestion. For instance, the top-ranked antibiotic returned by KART for the query “What antibiotic is used to treat *cystitis* caused by *Escherichia coli* for *pregnant women*?” was *ciprofloxacin*, which is in fact contraindicated in pregnancy. The reading of the associated publications should point out that such treatment must be avoided.

A major issue hindering the global use and circulation of CPGs is their format variability. The most frequently used formatting is based on unstructured narratives, or narratives structured with vernacular conventions. It is therefore crucial to transform CPGs into machine-readable formats, a process that usually requires the collaboration of both medical experts and software engineers [21], [50]. Indeed, few medical experts have the skills required to directly translate clinical knowledge into a computerized format. However, the medical entity normalization module and the formalization module aiming to facilitate the transformation of recommendations to a machine-interpretable format were not well perceived, since they generated more confusion than expected. In particular, for the normalization module, several search sequences were usually necessary before finding the relevant concept, since it often proposed too general or too specific concepts.

Our question-answering module could benefit from further improvements. We propose for instance to validate the recommendations. We could consider the validation as a binary task; this is to say to classify the recommendations as correct or wrong based on the biomedical literature. A possible method could be to compare the amount of available literature for a given antibiotic in a given clinical case, relative to all the other antibiotics for the same clinical case. This approach could also be useful to detect the need to update some clinical recommendations: the relative diminution of the amount of literature for a given antibiotic regarding the other antibiotics would reflect the potential emergence of alternative treatments. We could thus imagine running the automatic validation at regular intervals, in order to provide an alerting system. Regarding the normalization module, it has mainly raised the problem that it is difficult to reach the desired granularity to represent the entity of interest. A possible solution here would be to add hierarchical navigation functionalities, allowing the user to find parents or children of a given concept, as exemplified by the SNOCat categorizer which integrates a SNOMED browser developed by the Virginia-Maryland Regional College of Veterinary Medicine’s SNOCat. Alternatively, data inference engines could be used. Concerning the formalization module, we think that it should take into account the possibility to formalize more complex clinical recommendations, by adding for instance data relative to dosage or treatment duration. Indeed, such information is generally delivered by CPGs. However, for sake of simplicity for this first study, we did not consider such additional data. A formalization module able to handle more complex clinical recommendations would also allow the possibility to integrate recommendations developed by third parties, which complexity will obviously differ from our predefined structure. Finally, regarding the web application, the use of a cloud based web solution might be required if the system is used at larger scale.
