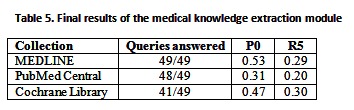# Correction: Assisted Knowledge Discovery for the Maintenance of Clinical Guidelines

**DOI:** 10.1371/annotation/b38c6896-f340-45cb-a9ca-ceff49330c3f

**Published:** 2013-05-10

**Authors:** Emilie Pasche, Patrick Ruch, Douglas Teodoro, Angela Huttner, Stephan Harbarth, Julien Gobeill, Rolf Wipfli, Christian Lovis

There was an error in Table 5. A correct version of the table is available here: 

**Figure:**